# Saliency Detection and Deep Learning-Based Wildfire Identification in UAV Imagery

**DOI:** 10.3390/s18030712

**Published:** 2018-02-27

**Authors:** Yi Zhao, Jiale Ma, Xiaohui Li, Jie Zhang

**Affiliations:** 1 AInML Lab, School of Electronics and Control Engineering, Chang’an University, Xi’an 710064, China; xiaohui.li@chd.edu.cn; 2 School of Automation, Southeast University, Nanjing 210009, China; jiale.ma@yahoo.com; 3 Shengyao Intelligence Technology Co. Ltd., Shanghai 201112, China; zhangj@hitrobotgroup.com

**Keywords:** UAV, wildfire, deep learning, saliency detection

## Abstract

An unmanned aerial vehicle (UAV) equipped with global positioning systems (GPS) can provide direct georeferenced imagery, mapping an area with high resolution. So far, the major difficulty in wildfire image classification is the lack of unified identification marks, the fire features of color, shape, texture (smoke, flame, or both) and background can vary significantly from one scene to another. Deep learning (e.g., DCNN for Deep Convolutional Neural Network) is very effective in high-level feature learning, however, a substantial amount of training images dataset is obligatory in optimizing its weights value and coefficients. In this work, we proposed a new saliency detection algorithm for fast location and segmentation of core fire area in aerial images. As the proposed method can effectively avoid feature loss caused by direct resizing; it is used in data augmentation and formation of a standard fire image dataset ‘UAV_Fire’. A 15-layered self-learning DCNN architecture named ‘Fire_Net’ is then presented as a self-learning fire feature exactor and classifier. We evaluated different architectures and several key parameters (drop out ratio, batch size, etc.) of the DCNN model regarding its validation accuracy. The proposed architecture outperformed previous methods by achieving an overall accuracy of 98%. Furthermore, ‘Fire_Net’ guarantied an average processing speed of 41.5 ms per image for real-time wildfire inspection. To demonstrate its practical utility, Fire_Net is tested on 40 sampled images in wildfire news reports and all of them have been accurately identified.

## 1. Introduction

Wildfire is a natural disaster, causing irreparable damage to local ecosystem. Sudden and uncontrollable wildfires can be a real threat to residents’ lives. Statistics from National Interagency Fire Center (NIFC) in the USA show that the burned area doubled from 1990 to 2015 in the USA. Recent wildfires in northern California (reported by CNN) have already resulted in more than 40 deaths and 50 missing. More than 200,000 local residents have been evacuated under emergency. The wildfires occur 220,000 times per year globally, the annual burned area is over 6 million hectares. Accurate and early detection of wildfire is therefore of great importance [1].

**Figure 1:**
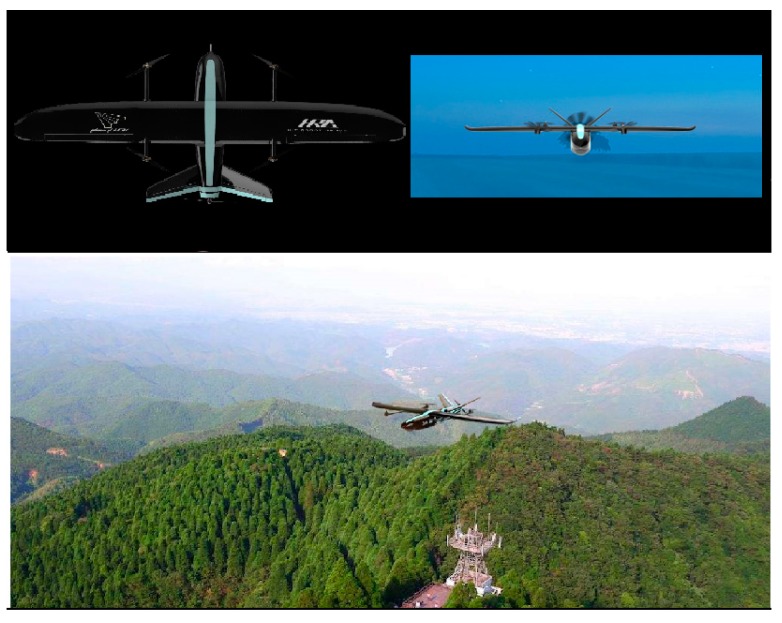
Forest Monitoring UAV PHECDA II developed by author of this paper.

Traditional wildfire detection is mostly based on human observation from watchtowers. Subject to the spatiotemporal limitation, it is inefficient. Unmanned aerial vehicles (UAVs or drones) have been increasingly used as surveillance tools [2]. UAVs equipped with global positioning systems (GPS) and ultrahigh resolution digital camera can provide HD aerial images with precise location information. Our previous work demonstrated that well organized UAV swarm is rapid, efficient and low-cost in conducting complicated agricultural applications [3]. Recent works also suggest that UAVs are quickly emerging implement in a variety of monitoring tasks [4,5,6]. The authors of this paper (Jie Zhang, Shanghai Shengyao Intelligence Technology Co. Ltd., Shanghai, China) developed a specialized UAV system (Figure 1) for forest and wildfire monitoring. The technique parameters of the forest monitoring UAV are presented in Table 1 below.

The main issue of this UAV monitoring system is that the whole system is operated by observers in surveillance center. Limited by the wireless transmission distance or the blind zone of GPRS/GSM network, real-time video transmission from the UAV can hardly be guaranteed. The surveyors often wait for the return of the UAV and examine its stored video. It is time consuming, labor intensive, and inefficient. Thus, an autonomous fire localization and identification system for this UAV is right now the top demand.

Early reported works on fire detection are generally based on sensor techniques. For example, heat and smoke detectors are mostly employed. These methods work properly in indoor environments. However, when applied to large outdoor areas, environmental factors can significantly impact their performance. Later, sensor network-based methods are reported effective in prediction rather than detection of wildfire [7], this is mainly because these methods depend on restricted data of relative temperature rise or wind speed in calculating the probability and intensity of fire. However, due to the limited measuring distance of each sensor, fine grained wildfire mapping over a large geographical area demands very dense deployment of sensors. It is difficult to implement in practice. Advanced sensor technology has been applied in fire detection: infrared sensors are used to capture the thermal radiation of fire, light detection and ranging system (LIDAR) [8] is employed to detect smoke by examining the backscattered laser light. Yet, these optical systems are also found sensitive to the varying atmospheric and environmental conditions: clouds, light reflections, and dust particles may lead to false alarms.

Satellite imagery is a common method for wildfire detection [9], but the long scan period and low flexibility make it difficult for early fire detection. Infrared (IR) thermographic cameras are used to generate thermal image of an area [10]. It can obtain reliable heat distribution data for fire detection. Yet most aerial IR thermographic imaging systems work on the wave band from 0.75 to 100 μm. They detect much less environmental information on this band. This information can also be very important, especially when flammable and combustible materials are presented. Also, limited by Nyquist Theorem, the recorded thermal image has lower spatial resolution than visible spectrum cameras. Moreover, thermographic systems are quite expensive with high maintenance costs.

Admittedly, visible spectrum CCD/CMOS imaging sensors are less sensitive to heat flux. Still they are capable of recording high resolution image of fire, smoke along with the surroundings. They are much cheaper than IR cameras and other type of sophisticated sensor system. Environmental changes have less influence on their performance. Additionally, the visible video-image-based methods combine well with existing monitoring systems: most UAVs and watchtowers have the visible spectrum camera equipped. These are the main reasons that many works using visible spectrum video and image to provide solid fire detection results [11,12,13].

Previous image processing-based fire detection research mostly relies on color and texture models. Chen proposed empirical models with experimental thresholds in detect flame pixels. Vipin proposed a fire pixel classification method using rule-based models in the RGB and YCbCr color space [14]. Angayarkkani and Radhakrishnan developed fuzzy rules in the YCbCr color space for fire image segmentation and detections [15]. Yuan developed a set of forest fire tracking algorithms including median filtering, color space conversion, and Otsu threshold [16]. These works are based on image processing techniques with handcrafted feature extractors, the results highly depend on the accuracy of the manually selected parameters.

Statistical and machine learning methods have been reported. Gaussian mixture model (GMM) is used in flame detection [17]. However, the use of empirical value of mixture number may not lead to the best results. SVM classifier is applied in [18]. It is noted that when commonly used feature descriptors like scale-invariant feature transform (SIFT) histogram of oriented gradients (HOG) are employed with these classifiers, the false alarm rate is not low enough.

Indeed, previous machine vision-based fire detection research are divided into two categories: flame detection and smoke detection [12]. Subject to the methods employed, these works are reliable on specific scene. Many are tested on short-range-shooting video or images with low infestation background. The volume of existing test benchmark is very small [19]. Up to this point, the fundamental difficulty has not yet been well addressed: how to build an adaptive classifier to identify complex fire features of different color, form, and texture from a varying cluttered background. Therefore, the objective of this paper is to provide an optimal wildfire feature learning model.

Deep convolutional neural networks (DCNN) have been reported to achieve state-of-art performance on a wide range of image recognition tasks [13], its architecture and learning scheme lead to an efficient extractor of complex, upper-level features which are highly robust to input transformations [20]. However, from the survey on UAV-based wildfire applications, deep learning technique has rarely been used in wildfire detections [21]. Until recently, deep learning has been reported in fire recognition in conference publications. Kim proposed an eight-layered CNN model in fire image classification [22]. The training image dataset are manually cropped and resized. Furthermore, the effect of key parameters and coefficients like dropout ratio, batch size, and learning rate have not been discussed.

### Problem Description

In this work, we try to present a complete fire localization and identification solution. To achieve this, there are some practical and technical problems must be solved:

1. Localization of fire area: First, unlike most contour prominent objects (humans, animals, cars, planes, buildings, etc.) wildfires have irregular form with very vague contours, their shape can vary dramatically with time. Yet, most machine vision-based detection methods are applied on objects with clear contour. Therefore, instead of find the whole fire boundary, localization of the core burring area can be more practical. Also, it is of great importance for further firefighting operation. As discussed in the previous section of this paper, most fire detection methods are tested on shoot-range shooting video or images. The effectiveness of these methods in localization of core fire area in high altitude aerial photographs remains uncertain.
2. Fixed training image size: Most deep learning models require input training image of fixed size (e.g., 128 × 128, 224 × 224). Yet, the most web-based image sources are of different sizes. In prevalent works, the reformatting of these images requires direct manual manipulation. Handcraft operations are slow, expensive, and inefficient. Worse still, wrapping, cropping, and direct resizing (ex., Gaussian pyramid and Laplacian pyramid method) may cause detail and feature loss or image blurring. In any case, this could lead to a poor DCNN training results [23]. Although method like Spatial pyramid pooling (SPP) [23] is used for DCNN to deal with varied size input images. Some disadvantages are notable: first, SPP is a high computational cost operation, according to the study in [24], the processing speed of SPP-net is considered unsuitable for real-time recognition. Secondly, the convolution layers preceding SPP cannot be updated during its fine tuning. This can be a major limit for a deeper network. Furthermore, high resolution aerial photography is often taken with a wide field of vision, these images usually contain much more background clutter information, which leads to high data redundancy, long calculating times, and poorer classification performance.
3. Limited amount of aerial view wildfire images: Generally, a DCNN requires a substantial amount of data to fully optimize the network’s parameters and weight values during its training procedure [25]. Insufficient training data will lead to overfitting and poor classification performance. The amount of wildfire aerial images available online is still very limited, thus a data augmentation necessary.

To address these problems, we proposed a new saliency detection-based segmentation method [26]. The method is tested effective in localization and extraction of the core fire area. In this way, most of the fire features can be conserved without severe feature loss. In addition, the method can be used to crop multiple fire regions into different fire images so that data augmentation can be achieved.

The rest of this paper is organized as follows. In Section 2, we present the new saliency detection method in localization and segmentation of core fire regions. In Section 3, the DCNN architecture is introduced in detail. The results and discussions are presented in Section 4 and Section 5 concludes the paper.

## 2. Saliency Segmentation and Image Dataset Formation

In this work, the original wildfire images were obtained from image searching engine of Google, Baidu, and the database of AInML lab in Chang’An University, The data source contains over 1500 images. These images are of varied size from 300 × 200 to 4000 × 3000 pixels. As our proposed DCNN architecture is implemented on Caffe [27]. The input images should be normalized into a standard LMDB database file. Thus, the image must be formatted to fixed size (e.g., 227 × 227 or 128 × 128). Therefore, a technical issue is raised: what measures should be taken to extract maximum fire features into the resized image dataset?

**Figure 2:**
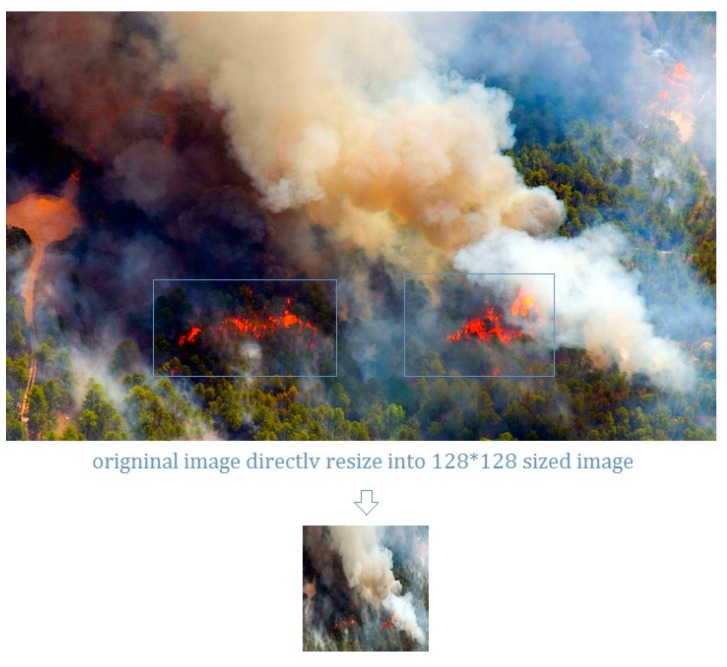
Direct resizing leads to detail loss.

As discussed in introduction, most prevalent methods use direct resizing or handcraft warping and cropping. These methods have some known disadvantages like detail loss and geometric distortion [23]. UAV imagery is usually taken with a wide-angle digital camera, the captured image contains highly redundant clutter information. When direct resizing is applied, some of the important fire features may be submerged into the background. For example, In Figure 2, the original image contains both flame and smoke features; however, after the resizing, the flame features have been submerged into the smoke.

**Figure 3:**
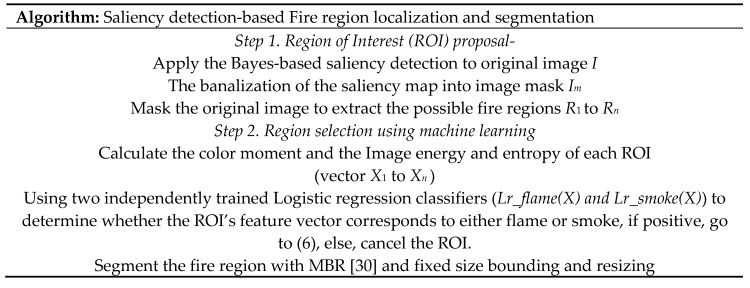
Saliency detection and logistic regression-based segmentation algorithm.

**Figure 4:**
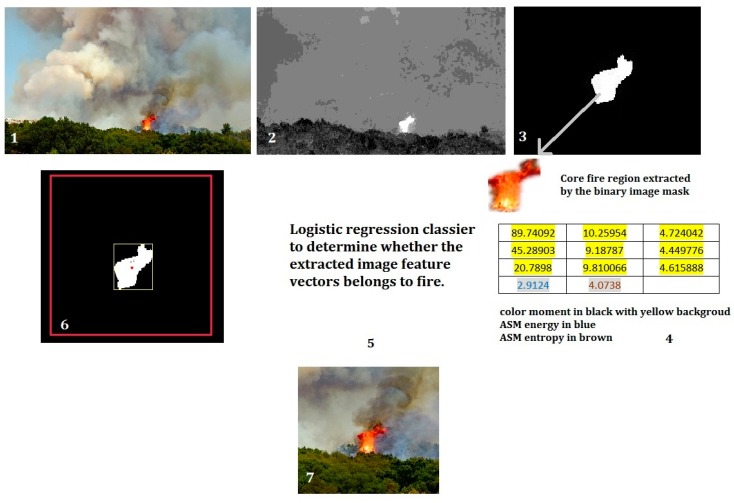
Example of the proposed fire localization and segmentation method.

In this work, we developed a new algorithm of wildfire localization and segmentation algorithm by combining saliency detection and logistic regression classifier (Figure 3). It can quickly locate the core fire region out of a complex background. An example of the method is illustrated in the Figure 4.

The algorithm can be divided into two phases: region proposal and region selection. In the first phase, saliency detection method is used to extract the region of interest (ROI, possible fire region in our case), we calculate the color and texture features of the ROIs. In the second phase, two logistic regression classifiers are used to determine whether the feature vector of ROIs belong to flame or smoke, if positive, we segment these regions.

Previous works suggests that color and texture are two effective and efficient features in image-based fire detection, therefore, we employ the value of color moment [28] as color feature descriptor while the image’s angular second moment (ASM) energy and entropy [29] as texture descriptors. The two groups of values are combined into the feature vector. The use of this feature vector can largely reduce the computational complexity.

The implementation of the algorithm are as follows.

### 2.1. Saliency Detection

Inspired by the primate’s vision system, saliency detection is highly effective in focusing on the core object from a complicated scene [31]. Early works on saliency detection have some common disadvantages like low resolution and blurred region boundary. Rahtu demonstrated in his work that the combination of saliency measure and conditional random field (CRF) model is valid in segmenting both still image and scenes from video sequences [26].

Rahtu’s Bayesian method uses a slide window to calculate the conditional probability of each pixel as its estimated saliency value, if the object’s feature strongly differs from the background, it obtained a high saliency value. The mechanism of the method is presented as follows: a slide window *W* is composed of both inner kernel *K* and border *B*. A pixel *x* in *W* can be either salient *H*_1_ *(in K)* or non-salient *H*_0_ *(in B)*. Accordingly, the probability for the each cases is *P (H*_1_*)* and *P (H*_0_*)*, suppose the kernel *K* contains the salient object, its feature distribution is therefore differs from the distribution of border B. Based on this assumption, the conditional feature distribution *p(F(x)|H*_1_*)* and *p(F(x)|H*_0_*)* can be estimated with the feature value *F(x)* computed at *x*. By Bayes’ definition of *P(A|B)*, we have the expression of $P\left(H_{1}|F\left(x\right)\right)$ written as

(1) $$P\left(H_{1}|F\left(x\right)\right)=\frac{p(F\left(x\right)|H_{1})P\left(H_{1}\right)}{p\left(F\left(x\right)|H_{0}\right)P\left(H_{0}\right)+p(F\left(x\right)|H_{1})P\left(H_{1}\right)}$$

Based on Equation (1), for each pixel in K, we use this estimated probability as its saliency level *S(x)*

(2) $$S\left(x\right)=P\left(H_{1}|F\left(x\right)\right)$$

This value shows the contrast level of the feature values in *K* and *B*. If the contrast between *K* and *B* is stronger, the saliency level of pixels in *K* is higher. In order to improve the robustness of the method, a Gaussian kernel is applied to smooth the histograms.

(3) $$\hat{p}\left(F\left(x\right)|H_{0}\right)=N\left(g\left(F\right)*h_{B}\left(F\right)\right)$$

(4) $$\hat{p}\left(F\left(x\right)|H_{1}\right)=N\left(g\left(F\right)*h_{K}\left(F\right)\right)$$

where *N* is the normalization operation, $h_{K}\left(F\right)\;$and $h_{B}\left(F\right)$ are the histogram respectively in *K* and *B*, g(F) is the Gaussian smoothing function. The authors of this paper applied this method in segmenting pests from its background, and we enhanced the segmentation details by adding edge-aware weightings [32].

### 2.2. Color Moment, ASM Energy, and Entropy

Color moment provides a measurement for color feature, it uses three central moments of an image’s color distribution. They are mean, standard deviation, and skewness. For example, an RGB image has three color channels, thus, three moments multiplied by three color channels makes nine moments in total. We define the *i*th color channel at the *j*th image pixel as *P_ij_.* The three color moments can be defined as

(5) $${\;E}_{i}=\;\overset{j=1}{\underset{N}{\sum }}\frac{1}{N}p_{ij}$$

(6) $$\sigma_{i}=\sqrt{\frac{1}{N}\overset{j=1}{\underset{N}{\sum }}{\left(p_{ij}-E_{i}\right)}^{2}}$$

(7) $$s_{i}=\sqrt{\frac{1}{N}\sum_{N}^{j=1}{\left(p_{ij}-E_{i}\right)}^{3}}$$

where *E* is the mean value, it is the average color value, *σ* stands for the standard deviation, it is calculated by the square root of color-distribution’s variance, *S* is the skewness, and it represents the asymmetry in the distribution.

The ASM energy and entropy are used to describe the texture feature of an image. They are expressed mathematically below

(8) $${\mathrm{ASM}}_{\mathrm{Energy}}=\;\underset{i,j}{\sum }(P{\left(i,j\;|\;d,\theta \right)}^{2}$$

(9) $${\mathrm{ASM}}_{\mathrm{Entropy}}=\;\underset{i,j}{\sum }(P(i,j\;|\;d,\;\theta )\;\log\left(P(i,j\;|\;d,\;\theta \right))$$

where *P* is the element of the ASM matrix, it stands for the occurrence probability that a gray value *i* and *j* of a pair pixels separated by distance *d* with an angle *θ*. The ASM energy (Equation (8)) is the square sum of all element values in ASM matrix. It reflects the uniformity of the gray level distribution and the roughness of the texture. If all the values of the matrix are nearly equal, the energy value is small; otherwise, the energy value is high. Similarly, ASM entropy is a measurement of non-uniformity or complexity of the texture in an image. A more dispersed distribution of ASM elements value leads to high entropy.

### 2.3. Logistic Regression Classifier

Logistic regression is one of the most employed binary classification method. As introduced in the previous section, the input of the classifier is the extracted ROI’s feature vector, expressed as *X =* [*x*_0_*, x*_1_*, x*_2_*, x*_3_······*x*_10_]*.* It is a vector of eleven dimensions, in which, *x*_0_ to *x*_8_ are the color moment, *x*_9_ is the ASM energy and *x*_10_ the ASM entropy. Accordingly, the trained weight’s vector should also be 11 dimensions as *W =* [*w*_0_*, w*_1_*, w*_2_*, w*_3······_*w*_10_]. We chose sigmoid as classifier function, the form of the classifier is

(10) $$h_{W}\left(X\right)=\;\sigma \left(W^{T}X\right)=\frac{1}{1\;+\;e^{-\left(W^{T}X\right)}}$$

Given an input feature vector *X* and the trained weight vector of *W*, the output value of this equation will situate between 0 and 1, if this value is above 0.5, we consider the feature vector belongs to positive class, otherwise, if the value is below 0.5, it is then negative. As the classification results are directly related to the weight vector, the aim of logistic regression is to find the optimal *W* in Equation (10). To achieve this, a gradient rise method is applied. First, based on Equation (10), the correspondent probability can be written as

(11) $$P\left(y|X;W\right)={\left(h_{W}\left(X\right)\right)}^{y}{\left(1-h_{W}\left(X\right)\right)}^{1-y}$$

Followed by a maximum likelihood estimation on W

(12) $$L\left(W\right)=\overset{m}{\underset{i=1}{\prod }}P\left(y^{\left(i\right)}|X^{\left(i\right)};W\right)=\overset{m}{\underset{i=1}{\prod }}{\left(h_{W}\left(X^{\left(i\right)}\right)\right)}^{y\left(i\right)}{\left(1-h_{W}\left(X^{\left(i\right)}\right)\right)}^{1-y\left(i\right)}$$

where *i* is the sample number of training feature vectors. For the convenience of calculation, Equation (12) is usually used in Logarithmic form. To gain the maximum value of *L,* we use the gradient ascent algorithm to update the value of W

(13) $$W_{j}=W_{j}+\alpha \;\nabla \left(\log L\left(w\right)\right)+\lambda X_{j}^{\left(i\right)}=\;W_{j}+\alpha \overset{m}{\underset{i=1}{\sum }}(y^{\left(i\right)}-h_{W}\left(X^{\left(i\right)}\right))X_{j}^{\left(i\right)}+\lambda X_{j}^{\left(i\right)}$$

where α is the learning rate, ∇(logL(w)) is the gradient of  logL(w), and it equals to $\overset{m}{\underset{i=1}{\sum }}(y^{\left(i\right)}-h_{W}\left(X^{\left(i\right)}\right))X_{j}^{\left(i\right)}$. $\lambda X_{j}^{\left(i\right)}$ is the disturbance term we add to avoid the overfitting problem.

In this work, two regression classifiers for flame and smoke feature are trained with two sets of 150 sampled flame and smoke image blocks, respectively. Stochastic gradient ascent method is applied during the training, thus the weights get updated in every training epoch. The training rate α is 0.001, and the coefficient of disturbance is set to 0.00001.

The reason for using two binary classifiers instead of one multiple-labeled classifier (e.g., Softmax classifier) is that we consider the feature of flame and smoke are not mutually independent. Notably, the gray level texture features of flame and smoke are often mixed. In this case, two independent binary logistic regression classifiers can be more effective.

As shown in Figure 4, the fire region shows the highest saliency value. After extraction by the saliency map, the core fire region’s image feature vector is calculated. The vector is then examined by the trained logistic regression classifier. With the positive results, minimum bounding rectangle (MBR) [30] method is used to locate the core fire region’s geometric center and crop the fire region into a standard sized image. This method keeps the full feature of fire (both flame and smoke) without distortion. This method has been applied to form the standard image dataset ‘UAV_Fire’. Furthermore, as demonstrated in Figure 4, this method can locate the core flame zone, which is considered highly practical in UAV wildfire inspection. More results can be found in the Section 4 of this paper.

## 3. The DCNN Model

In this work, we try to provide a more complete assessment of DCNN’s self-learning capability by comparing different architectures and evaluating the effects of the key parameters. DCNN is used as a classifier to determine whether wildfire happens in the UAV imagery. We developed a 15-layered DCNN model called ‘Fire_Net’. This section provides a precise and detailed description of the proposed DCNN model.

### 3.1. Overall Architecture

**Figure 5:**
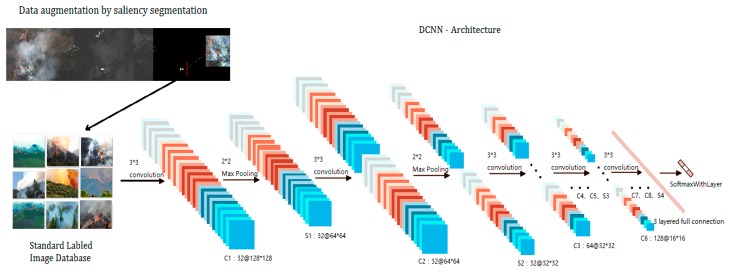
The overall architecture of ‘Fire_Net’.

The overall architecture is illustrated in Figure 5. The left part is the image saliency segmentation and image cropping stage. The right part is the core DCNN model. Inspired by Alexnet (eight-layered DCNN) [25], we proposed a deeper 15-layered DCNN model ‘Fire_Net’. Its structure is detailed in Table 2. With its self-learning structure, it can extract wildfire features from low to high level. The first 12 layers consist of 8 convolutional layers (Conv1–Conv8) and 4 max pooling (Pool1–Pool4). The convolutional layers extract the fire features from low to high level. The max pooling layers are used to capture deformable parts and reduce the dimension of the convolutional output. The last three fully connected layers (FC1, FC2, and IP3) capture complex co-occurrence statistics. A final classification layer summarize the previous abstracted high level features for the recognition of a given image. This architecture is appropriate for learning complex wildfire features from the training dataset. The complete schematic of the DCNN model can be found in Reference 1.

### 3.2. Fire_Net: The DCNN Model in Detail

- Input Layer: the input data are the saliency segmented image database in LMDB format, the standard size is 128 × 128 pixels. In the input layer, the color of the range of three channels of RGB is normalized to 0–1, so the proportion of 0.00390625 (1/256) is applied to the original value. During training, the batch size is 50 images, and the batch size in the validation phase is 10 images.
- Convolution layers: In Fire_Net, eight convolutional layers (Conv1–Conv8) are integrated, convolutional kernel size is defined 3 × 3, and sliding step length is 1. The weight initialization of parameter uses the Xavier way [33] and the bias is initialized with an experienced value. A rectified linear units (ReLU) activation function [34] follows each convolutional layer. The output of the ReLU function is then send to next convolution layer or pooling layer. The main reason of using ReLU instead of Tanh-Sigmoid as activation function is discussed as follows. When the input is very small or very large, the gradient of the sigmoid function can be near zero. This is called the ‘saturation’ effect. Comparatively, the ReLU function can be much faster in gradient descent-based training because it is a non-saturating function.
- Pooling layer: the objective of pooling layer is to reduce the data size and prevent over fitting. The kernel size of all pooling in this project is 2 × 2, the sliding step length is 2, and the max-pooling method [35] is applied.
- Full connection layer: FC1 and FC2 are the hidden layers, the number of neurons are 256 per layer, the weight initialization used Xavier mode [33]. Each neuron in the hidden layer uses a dropout technique to reduce the dependency between neurons, thus, the network’s overfitting can be reduced [36]. The activation function in full connection layer is also ReLU.
- Classification layer: the classification layer contains the output layer of the full connection neural network, a Softmax and cross entropy function. The number of the output layer neurons is two. Following the output layer neuron, a Softmax function [37] is applied, in this work, the Fire_Net model should be able to identify if the wildfire occurs. Thus, it becomes a binary classification problem (fire or non-fire).
  (14) $$\mathrm{Softmax}=\frac{\exp(a_{i})}{\sum_{j}\exp(a_{j})},$$
  where *i* corresponds to the number of classes, in this case, *i* equals to either 0 or 1 since it is a binary classification problem. The output of the Softmax function corresponds to the probability of the output class. The function is a monotonic function, thus, the output increases as the input increases. If the test image shows a dense wildfire feature, the output of the Softmax therefore corresponds to a higher value. The Softmax then adopted a cross-entropy loss regression to choose the outputs with the highest probability as the classification result.

### 3.3. Network Training

Fire_Net’s training is operated on a GPU of NVidia GeForce 840M. It is a low power 64-bit Maxwell architectured graphic processor for mobile applications. Thus, it is more appropriate to be used on a UAV. The optimal configuration of the Fire_Net’s training parameters are presented in the following section. As Caffe supports breakpoints observation, we observe the training error after each iteration in the terminal. Also, we save the training state and weight parameters at every 5000 iterations.

During the training, labeled images of size 128 × 128 pixels are given to the DCNN. The errors between actual output value and the desired value (label) are calculated. A stochastic gradient descent (SGD) method [24] is applied to train the DCNN. The SGD method optimizes coefficients and weights by computing derivatives after back-propagating the errors through the previous layers. This learning procedure is iterated until the training error is considered negligible.

## 4. Results and Discussion

### 4.1. Saliency Segmentation and Data Augmentation

**Figure 6:**
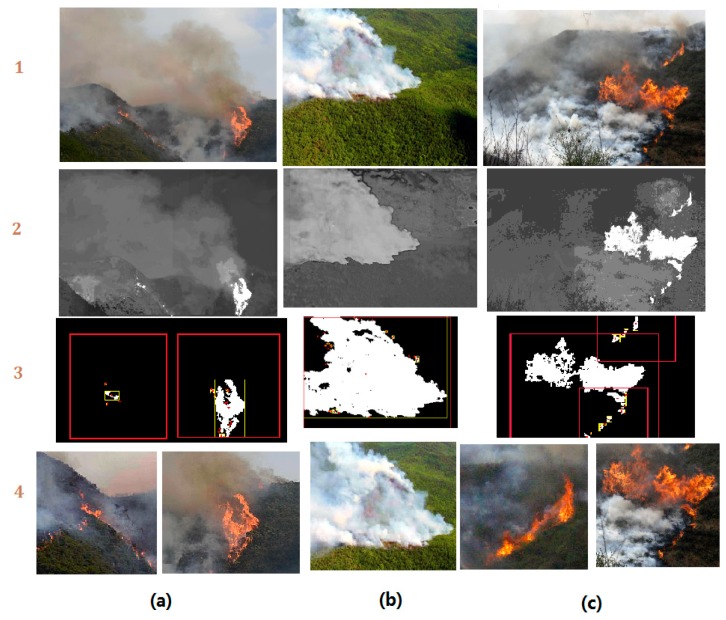
The saliency detection-based image segmentation: (**1**) Original image; (**2**) Saliency detection results; (**3**) ROI selection with logistic regression classifier and segmentation; (**4**) Segmented standard sized image for Fire_Net training. (**a**) Image with both flame and smoke features; (**b**) Image with only smoke feature; (**c**) Image with multiple fire regions.

As introduced in Section 2 of this paper, we used saliency detection-based segmentation method to locate the core fire region and segment them into standard sized images for Fire_Net training. The result is shown in Figure 6 below.

**Figure 7:**
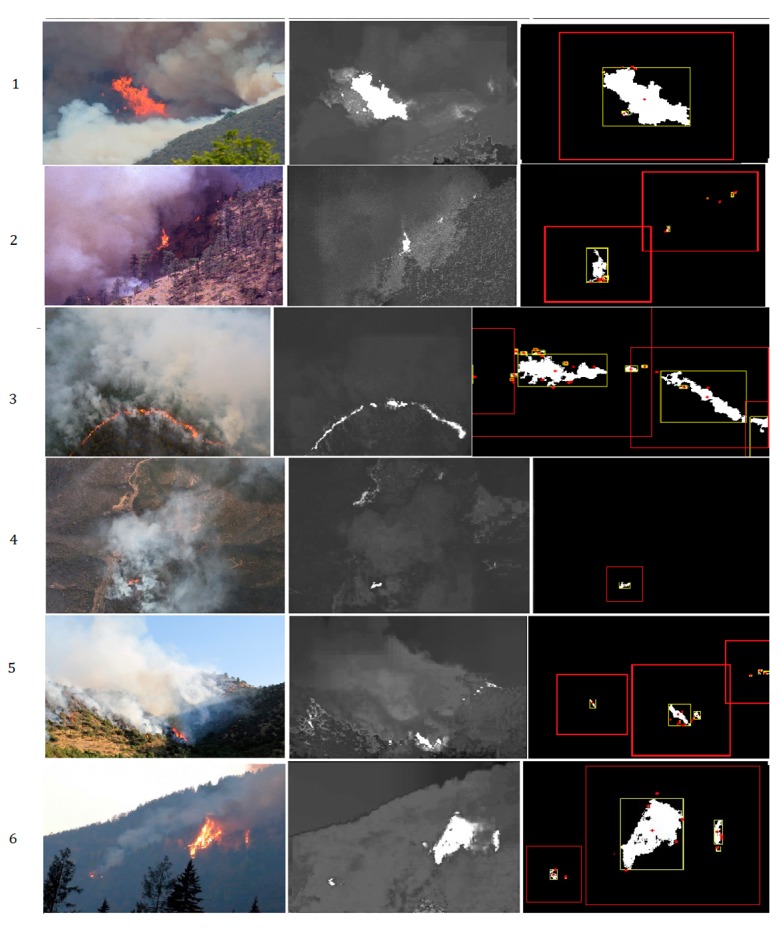
Examples of fire localization and segmentation method on several complex scenes.

As shown in Figure 6, the proposed saliency methods can efficiently locate the core fire regions in aerial images, even very tiny ignition zone (see Figure 6(a-3,c-3)) are well located. The segmented image contains a maximum fire feature without severe distortion or feature loss. Furthermore, in both Figure 6a,c, we used the proposed method to segment the original image into independent fire images and they all contain full fire features. This is how the saliency segmentation technique is adopted in augmentation of the original training image dataset. The augmentation of data helps to overcome the overfitting effect due to smaller training samples. More examples of test results are presented in the Figure 7.

The performance of the method is shown in Table 3. We tested the method on a set of selected 450 fire images with both flame and smoke features from the original image database. TP, FN, TN, FP refer to true positive, false negative, true negative, and false positive respectively. TPR is the true positive rate while FPR is false positive rate. It is found that the method achieved high accuracy in locating core fire areas in aerial images.

Finally, the segmentation method is applied on 1105 images with both flame and smoke features from the original image dataset, the rest of the images are not applied with this method. Because either these images are small in size or the flame and smoke features already covered most of the area. In this case, segmentation is not necessary. After data augmentation, the new image dataset contain over 3500 images. The detail of the augmented image database is presented in Table 4 below.

### 4.2. Fire_Net Parameter Optimization

The Fire_Net DCNN model includes a set of parameters such as the convolutional kernel size, the number of kernels, learning rate, dropout ratio, batch size, etc. Most of the parameters are initialized with experienced values from previous works [38]. After a full parameter tuning test, we find that some of the experienced value (input image size of 128 × 128, 128 convolution kernels size of 3 × 3, learning rate of 0.0001, convolution stride and pad of 1, max-pooling kernel size of 2, etc.) have indeed achieved optimum results in term of validation and test accuracy. Some of the experienced parameters does not lead to optimal results. Particularly, we find the value of dropout ratio and batch size are more sensitive regarding the model’s accuracy. Thus, we present the results of the test on the two parameters in Table 5 below.

As shown in Table 5, the optimal coefficient combination in Fire_Net is the batch size of 50 and the dropout ration of 0.5.

### 4.3. Model and Method Comparasion

**Figure 8:**
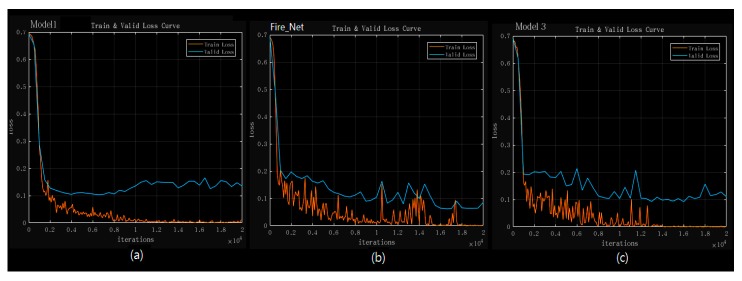
Training and validation loss curve of the three models: (**a**) Model 1; (**b**) Fire_Net; (**c**) Model 3. The curve is computed over an epoch of 20,000 iterations. The training loss is marked every 100 iterations while the validation loss is recorded every 500 iterations.

In order to further test the proposed method, we explored several types of DCNN architectures based on Fire_Net. Furthermore, we used both original image dataset and the augmented image dataset to evaluate the effects of the data augmentation to the model’s accuracy. The results are presented in Table 6 and the training–validation curve of Model 1–Model 3 is presented in Figure 8.

Both Figure 8 and Table 6 suggest that Fire_Net has an optimal performance in consideration of both speed and accuracy. In Figure 8, Fire_Net’s training and validation loss reduces to the smallest loss value. In Table 6, it is noted that the 20-layered Model 4 has also reached validation accuracy of 0.98, the same of Fire_Net, however, the average processing time for Model 4 is 1.63 times longer than Fire_Net. This is mainly due to the computation time cost in its added layers. Thus, Fire_Net is considered as a more efficient DCNN architecture in Fire recognition in terms of both validation accuracy and processing time. A more notable results in Table 6 is that we find all type of models trained by our augmented image database have shown better performance compared to those trained with original images. This is mainly because the deep architecture’s coefficients are optimized with sufficient training images while models trained with original image database suffers from lower training data quality. Furthermore, augmented database give the architecture more learning samples so that the coefficient and weights value become more optimal after the training. Original image dataset contains smaller amount of training image which could be easier to have an overfitting problem.

A standard test image dataset is used to evaluate Fire_Net’s recognition accuracy and generalization performance. Given an input test image, the convolution-pooling layers extract its high-level features and the full connection layer will determine whether the abstracted features correspondent to fire or not.

**Figure 9:**
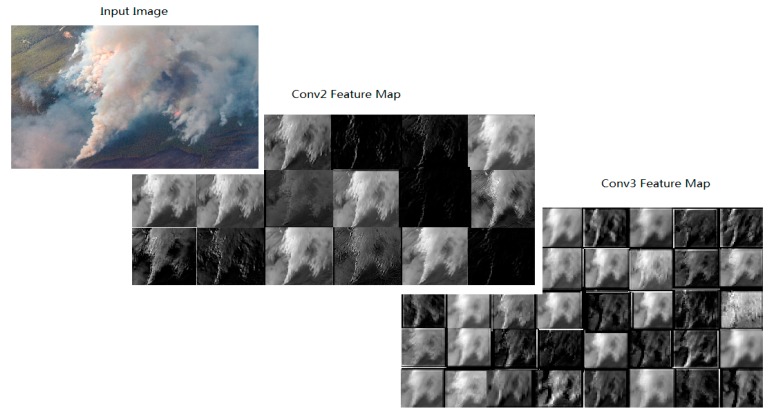
Visualization of feature maps after layer Conv2 and layer Conv3 in Fire_Net.

**Figure 10:**
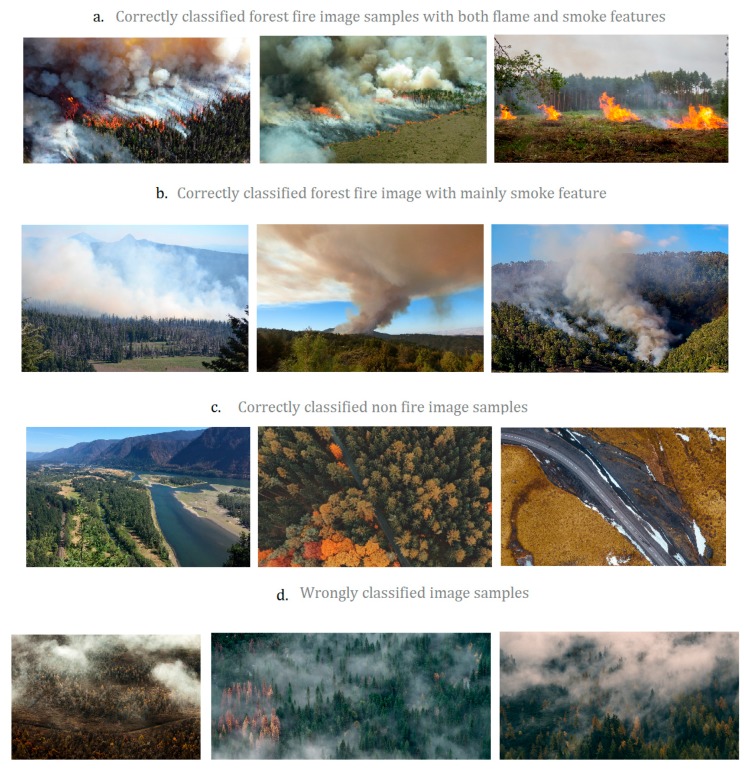
Samples of Fire_Net classification results. (**a**) Fire image of full fire features; (**b**) fire image with mainly smoke features; (**c**) no-fire images; (**d**) wrongly-classified images.

We visualized the feature maps after Conv1, Conv2, and Conv3 layers in Figure 9. It is well observed that the lower layers captures the low level features (texture, edge, or color), the higher layers response to higher level sparse features by eliminating irrelevant content. Some examples of Fire_Net recognition results are presented in Figure 10.

It is shown in Figure 10 that normal aerial images of wildfire (part a, part b) and negative image samples (part c) are correctly classified. The effectiveness of the model is justified. Still, it is admitted that Fire_Net worked inadequately on a rare case of ‘forest mist’ images (wrongly classified samples in part d). Fire_Net wrongly classified these forest mist images into fire case. The main reason is that the mist features are highly similar to smoke features in both color and texture. Images like 30 and 31 contain a very strong feature of smoke, even the human vision system can mistake it for fire. In these cases, sensor techniques are needed.

The following table shows the performance of Fire_Net on the test set of 512 images. The test set is composed by 256 fire image and 256 no-fire images. These images are randomly collected from online resources, they are not used in the training or validation phase. These images are not processed by our segmentation method. TPR is true positive rate while TNR is the true negative rate. It is exhibited in Table 7 that Fire_Net has a very low false negative rate.

A comparison of our models with prevalent machine vision classification methods is made. Since these methods are all trained and evaluated with the same training and testing data source of Fire_Net (‘UAV_fire’ as training dataset, the 512 randomly collected images as test dataset), the general classification accuracy can be a direct indicator of their performance.

The classification results by different methods are summarized in Table 8, it is shown that the ‘HOG + SVM’ solution is inadequate in fire detection. The HOG features are highly related with the contour and shape of the object while fire and smoke has no regular shape or contours. Deep belief net with a back-propagation neural network combines the advantage of unsupervised learning and supervised learning, unlike CNN, DBN cannot take a multi-dimensional image as input, images are turned into vectors to fit its input format, the image’s structural information is lost, which limited the image feature learning ability of DBN. The rest of the methods are different models based on CNN. Fire_Net outperformed the previous deep learning models.

**Figure 11:**
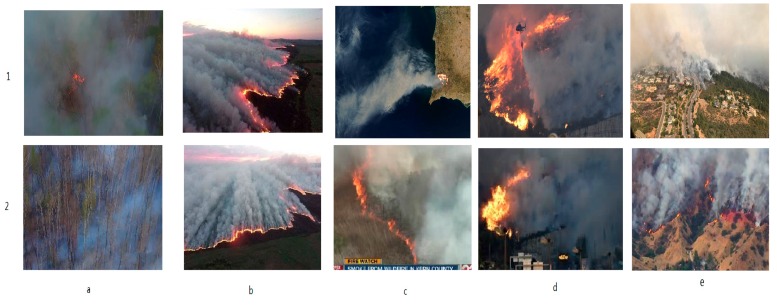
Examples of Fire_Net correctly-classified fire images from news reports. (**a**) Bilahe Forest fire, 2017; (**b**) Mongolia Forest fire, 2017; (**c**) South California wildfire and kern county wildfire, 2016; (**d**) Var wildfire, 2017; (**e**) La tuna and Napa valley wildfire, 2017.

We further evaluated Fire_Net’s generalization performance on a set of 40 sampled image from wildfire news reports. These samples are aerial images of Bilahe forest fire, in Noth China 2017(a); Mongolia Forest fire, in China, 2017(b); Envisat satellite image of wildfire in Southern California, USA. 2007(c1); Kern county wildfire, CA, US, 2016(c2); Var wildfire, south France, 2017(d); La Tuna and Napa Valley wildfire, CA, US, 2017(e); etc. All these samples are correctly classified as fire images: the output of Fire_Net ranges from 0.9765 to 1 for fire class, while for no-fire class, it is from 1.0064 × 10^−8^ to 0.0235. This indicates that strong fire features are presented in these images (see Figure 11).

## 5. Conclusions

In this paper, we demonstrated the effectiveness of using saliency detection and deep convolutional neural network in localization and recognition of wildfire in aerial images. The saliency detection method is used to locate core fire area and extract fire regions into multiple fire images. The proposed technique prevented severe feature loss due to direct resizing. Also, this technique significantly enriched the volume of the database. We also proposed a DCNN architecture named ‘Fire_Net’. It obtained satisfactory classification results. The pipeline of adopting saliency detection and deep learning in wildfire identification have not yet been reported previously, it is firstly proposed and verified in this work. Still, our method can be improved further. Regarding the wrongly classified forest mist images, IR sensors could be implemented in the decision making layers (full connection layers) of Fire_Net so that both image and sensor data can be used to determine whether fire occurs.

## Figures and Tables

**Table 1:** Technical parameters of the forest monitoring UAV PHECDA II.

Technical Parameters	Value
Wing span	2.7 m
Takeoff weight	18 to 25 kg (full load)
Maximum flight altitude	4000 m
Average cruising speed	90 km/h
Max flight speed	120 km/h
Endurance	1 h (pure battery powered)/6 h (gas–electric hybrid powered)
Digital camera equiped	FOV 94” 20 mm F2.8 4K-12 million pixels
Positioning system	GPS/GLONASS dual-module

**Table 2:** Definition of layers in ‘Fire_Net’.

Layer	Definition	Feature Maps	Kernel Size	Step
1	Conv1	32	3 × 3	1
2	Pool1	32	2 × 2	2
3	Conv2	32	3 × 3	1
4	Pool2	32	2 × 2	2
5	Conv3	64	3 × 3	1
6	Conv4	64	3 × 3	1
7	Conv5	128	3 × 3	1
8	Pool3	128	2 × 2	2
9	Conv6	128	3 × 3	1
10	Conv7	256	3 × 3	1
11	Conv8	256	3 × 3	1
12	Pool4	256	2 × 2	2
13	FC1(Full-Connection)	Number of neurons: 256		
14	FC2(Full-Connection)	Number of neurons: 256		
15	IP3(Softmax)	Output layer neurons: 2		

**Table 3:** Performance of the proposed localization and segmentation methods.

Image Type	190 Aerial View Fire Images Total ROIs Detected 384						260 Normal View Images Total ROIs Detected 581					
Performance	TP	FN	FP	TN	TPR	FPR	TP	FN	FP	TN	TPR	FPR
	269	12	5	98	95.7%	4.9%	421	38	9	113	91.7%	7.37%

**Table 4:** Description of original and augmented image database ‘UAV_Fire’.

Image Type	Original Image Database			Augmented Image Database ‘UAV_Fire’		
	UAV/Aerial/Remote-Sensing	Ordinary View	Total	UAV/Aerial/Remote-Sensing	Ordinary View	Total
Fire	197	435	632	462	1097	1559
No-fire	250	658	908	677	1325	2002
total	447	1093	1540	1139	2422	3561

**Table 5:** Different dropout ratio and batch size effects on validation accuracy.

Fire_Net Training Parameters			Different Batch Size Effects		Different Dropout Ratio Effects	
Learning Rate	Momentum	Max-Iteration	Batch Size	Validation Accuracy	Dropout Ratio	Validation Accuracy
0.0001	0.9	20000	16	0.952	0.2	0.95
0.0001	0.9	20000	32	0.958	0.3	0.946
0.0001	0.9	20000	48	0.969	0.4	0.967
0.0001	0.9	20000	50	0.972	0.5	0.98
0.0001	0.9	20000	64	0.97	0.6	0.972
0.0001	0.9	20000	128	--	0.7	0.955

**Table 6:** Effects of model architecture on performance and runtime.

Model Type Index	Architecture	Average Processing Speed	Training Dataset	Validation Accuracy	
Model 1	13-layered architecture:	31.9ms	Original image dataset	0.952	
	6xConv + 4xPooling:				
	Conv1-Conv2-Pool1-Conv3-Pool2-Conv4-Conv5-Pool3-Conv6-Pool4-FCx2-Softmax				
			Saliency-based augmented dataset	0.971	
Model 2	Fire_Net	41.5ms	Original image dataset	0.974	
			Saliency-based augmented dataset	0.98	
Model 3	15-layered architecture:	38.6ms	Original image dataset	0.972	
	8xConv + 4xPooling:				
	Conv1-Conv2-Pool1-Conv3-Conv4-Pool2-Conv5-Conv6-Conv7-Pool3-Conv8-Pool4-FCx2-Softmax				
			Saliency-based augmented dataset	0.978	
Model 4	20-layered architecture:	67.8ms	Original image dataset	0.977	
	11xConv + 6xPooling:				
	Conv1-Conv2-Conv3-Pool1-Conv4-Conv5-Pool2-Conv6-Pool3-Conv7-Conv8-Conv9-Pool4-Conv10-Pool5-Conv11-Pool6-FCx2-Softmax				
			Saliency-based augmented dataset	0.98	

**Table 7:** Performance of Fire Net on test dataset.

TP	FN	TN	FP	TPR	TNR	FNR	General Accuracy
253	3	249	7	98.8%	97.2%	0.12%	98% (502/512)

**Table 8:** Comparison of Fire_Net with other methods.

Models	Classifier	Accuracy
HOG + SVM	SVM	42.9%
Deep belief net + neural net	BPNN	87.2%
Kim’s CNN model^22^	Softmax	92.8%
Eight-layer CNN + Fisher vector	SVM	94.7%
AlexNet	Softmax	97.1%
Fire_Net	Softmax	98.0%

## Appendix A

The complete structure of Fire_Net:

## References

[1] Skala K., Dubravić A. (2008). Integrated System for Forest Fire Early Detection and Management. Period. Biol..

[2] Colomina I., Molina P. (2014). Unmanned aerial systems for photogrammetry and remote sensing: A review. ISPRS J. Photogramm..

[3] Li X., Zhao Y., Zhang J. A Hybrid PSO Algorithm Based Flight Path Optimization for Multiple Agricultural UAVs. Proceedings of the International Conference on TOOLS with Artificial Intelligence.

[4] Trasviñamoreno C.A., Blasco R., Marco Á., Casas R., Trasviñacastro A. (2017). Unmanned Aerial Vehicle Based Wireless Sensor Network for Marine-Coastal Environment Monitoring. Sensors.

[5] Gonzalez L.F., Montes G., Puig E., Johnson S., Mengersen K., Gaston K.J. (2016). Unmanned Aerial Vehicles (UAVs) and Artificial Intelligence Revolutionizing Wildlife Monitoring and Conservation. Sensors.

[6] Lelong C.C.D., Burger P., Jubelin G., Bruno R., Sylvain L., Frederic B. (2008). Assessment of Unmanned Aerial Vehicles Imagery for Quantitative Monitoring of Wheat Crop in Small Plots. Sensors.

[7] Hefeeda M., Bagheri M. (2009). Forest Fire Modeling and Early Detection using Wireless Sensor Networks. Ad Hoc Sens. Wirel. Netw..

[8] Wulder M., White A., Alvarez J.C., Han F., Rogan T., Hawkes B. (2009). Characterizing boreal forest wildfire with multi-temporal landsat and lidar data. Remote Sens. Environ..

[9] Li Z., Nadon S., Cihlar J. (2000). Satellite detection of Canadian boreal forest fires: Development and application of the algorithm. Int. J. Remote Sens..

[10] Katayama H., Naitoh M., Suganuma M., Harada M., Okamura Y., Tange Y. (2009). Development of the Compact InfraRed Camera (CIRC) for wildfire detection. SPIE Opt. Eng. Appl..

[11] Jakovcevic T., Braovic M., Stipanicev D., Krstinic D. Review of wildfire smoke detection techniques based on visible spectrum video analysis. Proceedings of the International Symposium on Image & Signal Processing & Analysis.

[12] Çetin A.E., Dimitropoulos K., Gouverneur B., Grammalidis N., Günay O., Habiboglu Y.H. (2013). Video fire detection—Review. Digit. Signal Process..

[13] Lecun Y., Bengio Y., Hinton G. (2015). Deep learning. Nature.

[14] Vipin V. (2012). Image processing based forest fire detection. Int. J. Emerg. Technol. Adv. Eng..

[15] Angayarkkani K., Radhakrishnan N. (2010). An Intelligent System for Effective Forest Fire Detection Using Spatial Data. Int. J. Comput. Sci. Inf. Secur..

[16] Yuan C., Liu Z., Zhang Y. UAV-based forest fire detection and tracking using image processing techniques. Proceedings of the International Conference on Unmanned Aircraft Systems IEEE.

[17] Töreyin B.U., Dedeoğlu Y., Güdükbay U., Cetin A.E. (2006). Computer vision based method for real-time fire and flame detection. Pattern Recognit. Lett..

[18] Byoung C.K., Kwang-Ho C., Jae-Yeal N. (2009). Fire detection based on vision sensor and support vector machines. Fire Saf. J..

[19] Toulouse T., Rossi L., Akhloufi M., Celik T. (2015). Benchmarking of wildland fire colour segmentation algorithms. Image Process. IET.

[20] Girshick R., Donahue J., Darrell T., Malik J. Rich feature hierarchies for accurate object detection and semantic segmentation. Proceedings of the Conference on Computer Vision and Pattern Recognition.

[21] Yuan C., Zhang Y., Liu Z. (2015). A survey on technologies for automatic forest fire monitoring, detection. Can. J. For. Res..

[22] Kim S., Lee W., Park Y.S., Lee H.W., Lee Y.T. Forest fire monitoring system based on aerial image. Proceedings of the International Conference on Information and Communication Technologies for Disaster Management.

[23] He K., Zhang X., Ren S., Sun J. (2014). Spatial Pyramid Pooling in Deep Convolutional Networks for Visual Recognition. IEEE Trans. Pattern Anal. Mach. Intell..

[24] Girshick R. Fast R-CNN. Proceedings of the IEEE International Conference on Computer Vision IEEE.

[25] Krizhevsky A., Sutskever I., Hinton G.E. ImageNet classification with deep convolutional neural networks. Proceedings of the International Conference on Neural Information Processing Systems.

[26] Rahtu E. Segmenting Salient Objects from Images and Videos. Proceedings of the 11th European Conference on Computer Vision.

[27] Jia Y., Shellamer E., Donahue J., Karayev S., Long J., Girshick R., Guadarrama S., Darrell T. Caffe: Convolutional Architecture for Fast Feature Embedding. Proceedings of the ACM International Conference on Multimedia.

[28] Huang Z.C., Chan P.P.K., Ng W.W.Y., Yeung D.S. Content-based image retrieval using color moment and Gabor texture feature. Proceedings of the International Conference on Machine Learning and Cybernetics.

[29] Ortiz A., Górriz J.M., Ramírez J., Salas-González D., Llamas-Elvira J.M. (2013). Two fully-unsupervised methods for MR brain image segmentation using SOM-based strategies. Appl. Soft Comput. J..

[30] Kwak E., Habib A. (2014). Automatic representation and reconstruction of DBM from LiDAR data using Recursive Minimum Bounding Rectangle. J. Photogramm. Remote Sens..

[31] Itti L., Koch C., Niebur E. (1998). A Model of Saliency-Based Visual Attention for Rapid Scene Analysis. IEEE Trans. Pattern Anal. Mach. Intell..

[32] Zhao Y., Li X. Edge-Aware Weighting Enhanced Saliency Segmentation of Pests Images. Proceedings of the International Conference on Computational Science and Computational Intelligence IEEE.

[33] Glorot X., Bengio Y. (2010). Understanding the difficulty of training deep feedforward neural networks. J. Mach. Learn. Res..

[34] Nair V., Hinton G.E. Rectified Linear Units Improve Restricted Boltzmann Machines. Proceedings of the International Conference on Machine Learning.

[35] Giusti A., Dan C.C., Masci J., Gambardella L.M., Schmidhuber J. Fast image scanning with deep max-pooling convolutional neural networks. Proceedings of the IEEE International Conference on Image Processing.

[36] Srivastava N., Hinton G., Krizhevsky A., Sutskever I., Salakhutdinov R. (2014). Dropout: A simple way to prevent neural networks from overfitting. J. Mach. Learn. Res..

[37] Tüske Z., Tahir M.A., Schlüter R., Ney H. Integrating Gaussian mixtures into deep neural networks: Softmax layer with hidden variables. Proceedings of the IEEE International Conference on Acoustics, Speech and Signal Processing.

[38] Ren S., He K., Girshick R., Sun J. (2015). Faster R-CNN: Towards Real-Time Object Detection with Region Proposal Networks. IEEE Trans. Pattern Anal. Mach. Intell..

